# The GWAS-MAP|ovis platform for aggregation and analysis of genome-wide association study results in sheep

**DOI:** 10.18699/VJGB-22-46

**Published:** 2022-07

**Authors:** A.V. A.V. Kirichenko, A.S. Zlobin, T.I. Shashkova, N.A. Volkova, B.S. Iolchiev, V.A. Bagirov, P.M. Borodin, L.С. Karssen, Y.A. Tsepilov, Y.S. Aulchenko

**Affiliations:** Kurchatov Genomic Center of ICG SB RAS, Novosibirsk, Russia; Kurchatov Genomic Center of ICG SB RAS, Novosibirsk, Russia; nstitute of Cytology and Genetics of the Siberian Branch of the Russian Academy of Sciences, Novosibirsk, Russia; Federal Research Center for Animal Husbandry named after Academy Member L.K. Ernst, Dubrovitsy, Moscow region, Russia; Federal Research Center for Animal Husbandry named after Academy Member L.K. Ernst, Dubrovitsy, Moscow region, Russia; Federal Research Center for Animal Husbandry named after Academy Member L.K. Ernst, Dubrovitsy, Moscow region, Russia; Institute of Cytology and Genetics of the Siberian Branch of the Russian Academy of Sciences, Novosibirsk, Russia; PolyKnomics BV, ‘s-Hertogenbosch, The Netherlands; Kurchatov Genomic Center of ICG SB RAS, Novosibirsk, Russia Novosibirsk State University, Novosibirsk, Russia; Kurchatov Genomic Center of ICG SB RAS, Novosibirsk, Russia

**Keywords:** genome-wide association study, marker-based breeding, sheep, database, web interface, полногеномное исследование ассоциаций, маркер-ориентированная селекция, овцы, база данных, веб-интерфейс

## Abstract

In recent years, the number of genome-wide association studies (GWAS) carried out for various economically important animal traits has been increasing. GWAS discoveries provide summary statistics that can be used both for targeted marker-oriented selection and for studying the genetic control of economically important traits of farm animals. In contrast to research in human genetics, GWAS on farm animals often does not meet generally accepted standards (availability of information about effect and reference alleles, the size and direction of the effect, etc.). This greatly complicates the use of GWAS results for breeding needs. Within the framework of human genetics, there are several technological solutions for researching the harmonized results of GWAS, including one of the largest, the GWAS-MAP platform. For other types of living organisms, including economically important agricultural animals, there are no similar solutions. To our knowledge, no similar solution has been proposed to date for any of the species of economically important animals. As part of this work, we focused on creating a platform similar to GWAS-MAP for working with the results of GWAS of sheep, since sheep breeding is one of the most important branches of agriculture. By analogy with the GWAS-MAP platform for storing, unifying and analyzing human GWAS, we have created the GWAS-MAP|ovis platform. The platform currently contains information on more than 34 million associations between genomic sequence variants and traits of meat production in sheep. The platform can also be used to conduct colocalization analysis, a method that allows one to determine whether the association of a particular locus with two different traits is the result of pleiotropy or whether these traits are associated with different variants that are in linkage disequilibrium. This platform will be useful for breeders to select promising markers for breeding, as well as to obtain information for the introduction of genomic breeding and for scientists to replicate the results obtained.

## Abstract (ru)

В последние годы увеличивается количество полногеномных исследований ассоциаций (ПГИА, GWAS), проведенных для различных экономически важных признаков животных. Результаты этих исследований представлены в виде суммарных статистик, которые можно использовать для изучения генетического контроля экономически важных признаков сельскохозяйственных животных, в том числе и при разработке методик маркер-ориентированной селекции. В большинстве случаев ПГИА сельскохозяйственных животных не соответствуют общепринятым в области исследований генетики человека стандартам формата публикаций результатов ПГИА в виде суммарных статистик (наличие информации об эффекторном и референсном аллелях, значение и направление эффекта и др.). Это существенно затрудняет использование суммарных статистик для нужд селекции. В области исследований генетики человека имеется несколько технологических решений для анализа результатов ПГИА, в том числе одно из самых крупных – платформа GWAS-MAP. Для других видов живых организмов, включающих и экономически важных сельскохозяйственных животных, подобных решений нет. В настоящей работе мы сфокусировались на создании схожей платформы для работы с суммарными статистиками ПГИА различных признаков овец, так как овцеводство в последнее время становится все более актуальной областью сельского хозяйства. По аналогии с платформой GWAS-MAP для хранения, унификации и анализа GWAS человека мы создали платформу GWAS-MAP|ovis. На сегодняшний день платформа содержит информацию о более чем 34 млн ассоциаций между вариантами геномной последовательности и признаками мясной продуктивности. Платформа может быть использована и для проведения анализа колокализации – метода, который позволяет установить, является ли ассоциация определенного локуса с двумя разными признаками результатом плейотропии или же данные признаки ассоциированы с разными вариантами, которые находятся в неравновесии по сцеплению. Эта платформа будет полезна как селекционерам для выбора перспективных маркеров для селекции (эффекты и аллели различных маркеров, влияющих на изучаемые признаки), так и для ученых, ведущих исследования в области генетики овец.

## Introduction

A genome-wide association study (GWAS) is modern technique to detect genome loci associated with different traits both quantitative and binary ones. GWASs have been widely used in human and animal genetics (Visscher et al., 2017). Since 2007, an annual increase of GWAS-based investigations to search for trait-associated loci has been observed. A GWAS results in the so-called summary statistics, a text tables where each line is a result of association analysis between a studied trait and a single nucleotide polymorphism (SNP). In breeding, such summary statistics can be used to devise marker-assisted and genomic selection models and identify target candidates for genome editing. Thanks to the new-generation technologies of genome-wide genotyping and next-generation sequencing, modern GWASs enable one to study hundreds of thousands and oftentimes millions of SNP associations with dozens of traits in huge samples including some tens thousands of animals. In other words, GWAS summary statistics are huge datasets (Visscher et al., 2017), whose processing is far from being trivial and requires, apart from their collection, a platform to provide proper quality control, storage, access and processing tools.

In the field of human genetics, such a platform, known as GWAS-MAP (Shashkova et al., 2020), serves for unification, storing and analysis of millions of associations for thousands of human traits. However, to our knowledge, there is still no such a solution even for a single kind of livestock farming. There are only databases to process quantitative trait loci significantly associated with studied traits. The biggest of such databases is AnimalQTLdb that contains information on quantitative-trait loci for different kinds of livestock (Hu et al., 2013). There are similar solutions for particular kinds of animals such as the iSheep database that provides access to the known quantitative-trait loci, genotyping and sequencing data of sheep (Wang et al., 2021). Since none of the mentioned solutions provides opportunities for storing, unification and analysis of GWAS summary statistics, the objective of the presented study was to create such a platform for sheep.

Sheep breeding is an important branch of animal farming with mutton being its most popular product. Since 2013, more than 20 GWASs of economically important sheep traits have been known. Here, S. Bolormaa et al. (2016) is of particular note being the biggest GWAS for a meat productivity traits in sheep. The authors investigated a cohort of more than 10,000 animals to study information on 56 different genetic traits related to meat productivity and found more than 70 significantly associated SNPs included in 23 loci.

Here we present the GWAS-MAP|ovis platform that is analogous to GWAS-MAP and can be used for aggregation, unification, storing and analysis of the GWAS summary statistics of the economically important traits affecting meat production in sheep. Currently, the platform contains more than 34 mln associations for 80 such traits. Breeders may find it useful for selection based on the effects of the alleles associated with studied traits and on perspective selection markers. It may also be useful for the scientists investigating sheep genetics. In particular, the platform enables for GWAS quality control; search for significantly associated SNPs; GWAS results visualization and colocalization analysis.

## Materials and methods

Developing the GWAS-MAP|ovis platform. GWAS results are commonly summary statistics being big text tables in which a single line is the results of association analysis to match a studied trait and SNP. Summary statistics can include different columns such as SNP identifier (if any); SNP position in a physical genome map (chromosome and position), SNP imputation or genotyping quality control metrics; effective and reference alleles; effective allele fre- quency; SNP effect estimation; standard error and p- value; sample size and other technical variables specific for particular research. The minimum set of the variables necessary for summary statistics processing includes an SNP identifier, effective and reference alleles, effect estimation and a standard error. Without loss of information, the standard error can be replaced by either the p-value or by z-statistic. Since the number of SNPs is huge, the size of the file containing a summary statistics table can reach several gigabytes in a case the number of characterized SNPs exceeds 20 million

**Fig. 1.**
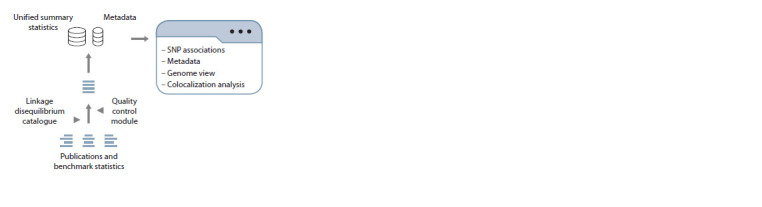
GWAS-MAP|ovis f low chart.

The GWAS-MAP platform (Shashkova, 2020; Shashkova, Aulchenko, 2020) to store GWAS results in the human was used as a basis to design the solution in question. For presentation and visualization of genetic data, the PheLiGe web interface was introduced (Shashkova et al., 2021). The flow chart of GWAS-MAP|ovis can be seen in Fig. 1.

To store and process data, GWAS-MAP uses two database management systems, ClickHouse and PostgreSQL. While ClickHouse serves to store matched summary statistics, PostgreSQL is used to store the metadata describing the sources summary statistics come from, a GWAS dataset and data analysis features. They may include e. g., a reference to the publication data come from; trait name; description of the model used for analysis; the number of studied animals, etc. Their full list is available in Supplementary material 11. The two systems are deployed in an LXC container and operate using an Ubuntu 18.04 operating system image.

One of the key steps in setting up the database was creating a linkage disequilibrium catalogue and the so-called reference table (see the description below). To provide convenient access to the database and enable data visualization as interactive graphs, the PheLiGe-ovis web interface was deployed in another LXC container and operated using the same operating system image. The web interface is available at https://pheligeovis.icgbio.ru/.

Supplementary Materials are available in the online version of the paper: http://vavilov.elpub.ru/jour/manager/files/Suppl_Kirichenko_engl.pdf


The designed platform having two databases to store summary statistics and metadata and a web interface was called GWAS-MAP|ovis and is available via public (web) and private (ssh) interfaces. The general principles of how to operate its web interface and main tools are described in the on-line assistant program, whose window pops up when pressing the red button under the Associations menu item in the top right corner of the platform’s main page.

Linkage disequilibrium catalogue is an SNP list that contains the following information for each included SNP: identifier (rs_id); chromosome and position (base on the OAR_V3.1 genome assembly); effective and reference allele, and effective allele frequency in referent population. To create the catalog were used the genome data of a reference population (N = 96) including 18 Romanov breed sheep, 6 Katahdin sheep, 10 argali, 48 F1 hybrids from crossing F1 (Romanov and argali) and Romanov breeds, and 14 F1 hybrids from crossing Romanov and Katahdin breeds. All the animal had been genotyped using Infinium® HD SNP Bead-Chip (606,060 ОНП, Illumina Inc., San Diego, CA, USA) and used for replication in our recent paper (Zlobin et al., 2021). Preparing the reference table involved removing all duplicated and monomorphic SNPs; the alleles were placed in lexicographic order; sex chromosome data were removed. For the time being, the disequilibrium catalogue contains information on 523.578 SNPs. Their autosomal distribution can be found in Supplementary material 2 and their minor allele frequencies in Supplementary material 3.

The linkage disequilibrium catalogue is files containing the genotypes of the reference population animals for every SNP included in the reference table. The files are in binary format PLINK (.bed, .bim and .fam). The catalogue is used to calculate the linkage disequilibrium between SNPs for finding a proxy SNP in the PheLiGe-ovis web interface.

Module of unification and quality control GWAS results. This module is used to download summary statistics in the database. Since these files are obtained from different sources and often have different formats, they have to be unified first. At this stage, matches between rs_id and the position in the chromosome and between the effective and reference alleles are checked

As the next stage, the summary statistics pass quality control that includes three stages: (1) comparison of GWAS allele frequencies against those in the linkage disequilibrium catalogue; (2) checking effect-size distribution, and (3) verifying if the p-value corresponds to z-statistic (effect estimation divided by a standard estimation error). The module generates an html report that can be used to detect unification and quality-control errors. If data pass the control, the summary statistics are stored in the platform’s database.

## Results and discussion

Content management

To fill the database, a search for published summary statistics was carried out in an early published database of the QTL and genes associated with meat productivity traits in sheep (Zlobin et al., 2019). Out of 46 publications analyzed, only S. Bolormaa et al. (2016) provided access to genome-wide summary statistics. Before being loaded in the GWAS-MAP|ovis database, all the data were unified and they passed the quality control. As a result, 488,074 associations for each of the 56 traits listed in the paper were downloaded in

In addition to the data provided by S. Bolormaa et al. (2016), the GWAS-MAP|ovis database also contains the summary statistics obtained from the Russian sheep population (N = 50–108) early used for replication analysis (Zlobin et al., 2021). This summary statistics described 7 composite indices related to the meat productivity measured for three different time periods (6, 42 and 90 days after birth) and an animal’s weight also measured for three time periods (for more details, see Supplementary material 4). As a result, the platform’s database has accumulated information on more than 34 million SNP associations for 80 different traits related to meat productivity in sheep. The available dataset and its short description are presented in the Table

**Table 1.**
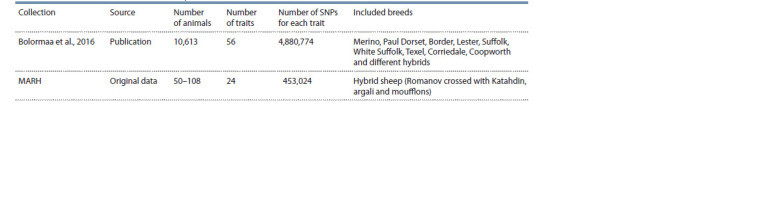
Data collections included in the GWAS-MAP|ovis database

Pleiotropy analysis

One of the advantages of the designed platform is that it can be used to perform colocalization analysis (SMR-𝜃) (Momozawa et al., 2018), which enables one to compare the association patterns of a particular genome locus for different traits and make conclusions whether this locus has a pleiotropic effect on a certain trait. In a nutshell, in presence of this effect, the ratio of estimated SNP effects on two traits in a studied locus is expected to be insignificant. However, if the association patterns are different i. e., the effect ratio changes significantly from one SNP to another, it is most likely that the locus contains different functional polymorphisms for each of the traits and they are being in linkage disequilibrium.

**Fig. 2.**
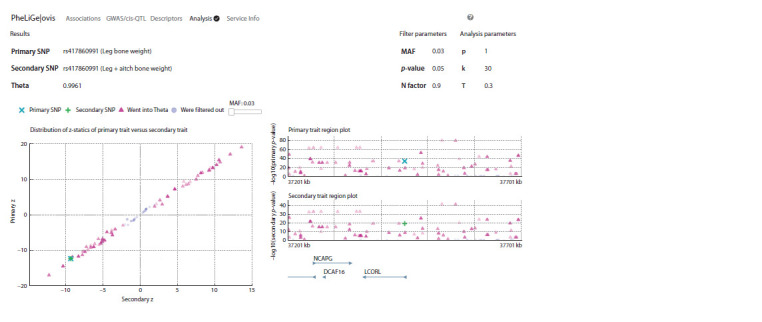
Results of the colocalization analysis performed using the GWAS-MAP|ovis platform The left part is a joint distribution of z-statistics for a main (leg bone weight, LEGBONE, X-axis) and secondary (leg + aitch bone weight, BONE, Y-axis) traits. The right part is regional association graphs for each of the traits (LEGBONE and BONE, respectively). The θ is indicated in the top left corner over the graph.

Statistic 𝜃 is a weighted correlation, whose computation requires information on 𝑝-values and an effect direction. The high absolute value (e. g., |𝜃| > 0.7) means a locus has a pleiotropic effect on investigated traits. If 𝜃 is positive, the SNPs of the locus have a similar effect direction towards the traits, and a divergent one if negative. An example of colocalization analysis is presented in Fig. 2.

**Fig. 3.**
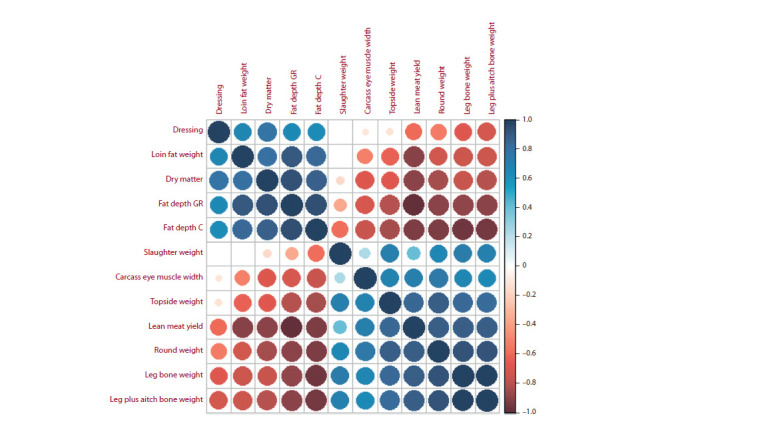
Results of colocalization analysis for SNP rs401834107 for 12 original traits, from (Bolormaa et al., 2016). The red color marks negative 𝜃-values, the blue – positive ones. The brighter the color and bigger the circle, the closer is the absolute value to one.

In the framework of the presented study, colocalization analysis was carried for the most significantly associated SNP of the LCORL1 gene in the data by (Bolormaa et al., 2016). For SNP rs401834107, 12 traits of genome-wide significance level ( p-value <5E–07) associated with this locus were indicated. The SMR-𝜃 method was applied to compare association patterns for all the 12 traits, the results are demonstrated in Fig. 3. The heat map characterizing 𝜃-values shows the traits form two clusters with different effect correlation directions, which means the locus had a pleiotropic effect inside each of the clusters. At the same time, the same strong effect was observed between traits from different clusters with divergent SNP effect on the traits (negative 𝜃 value). It is noteworthy that the slaughter weight trait was a dropout since some of its 𝜃-values turned out to be low (possible pleiotropy absence). However, due to the pleiotropic effect the locus had on the other traits, the low 𝜃-values in this particular case can be considered either as a statistic artifact or as a value produced due to insignificant analysis capacity.

Thus, it has been found that locus rs401834107 had a pleiotropic effect on at least 12 different traits associated with meat production in sheep. The presented data is an example of a first in the world colocalization analysis performed using sheep GWAS results.

Searching for candidate DNA markers for marker-assisted selection

In addition to the described pleiotropy analysis, another advantage of the GWAS-MAP|ovis platform is that it allows one to use previously loaded summary statistics to search for candidate markers to perform marker-assisted selection (MAS). In the presented study, such a search for the hot-carcass-weight (HCWT) trait was performed to estimate the markers’ predictive potential in a Russian sheep cohort. To begin with, a clumping procedure was carried out. To do this, the private (ssh) interface was used to select the summary statistics of interest and set up their significant threshold of p-value < 1E–07. Four independent loci were detected (rs406365427, rs423487570, rs161042491, rs427891980). A table containing information on SNP with the highest associations for each of the loci, their chromosomes, positions, effector and reference alleles and p-values was formed for further analysis.

The found loci were used to estimate the breeding value (BV) of 94 animals (Zlobin et al., 2021) to be the backcross hybrids of Romanov and argali sheep. Their BV was estimated as:

**Formula:**
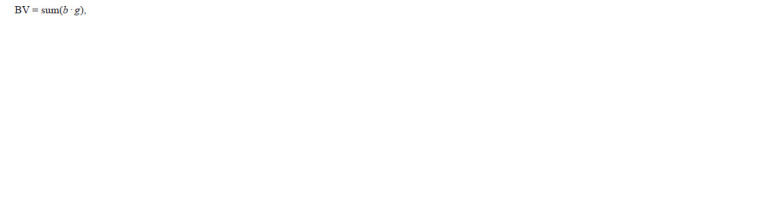
Formula

where b is the effect of the effective allele of the most associated SNP in every locus; g is the genotypes of 94 animals for that SNP encoded as 0, 1, 2 in relation to the effective allele’s quantity

Cases then underwent linear regression to find how significant the estimated BVs were for predicting a species’ phenotype. As a trait, an animal’s weight in six days after birth was considered. The used model was p ~ BV + cov, where p is a phenotype’s value; cov is the covariates including information about animal gender, yield number, and two first main components of their kinship matrix. As a result, the estimated BV was found to be significantly associated ( p-value = 0.03) with the trait, which confirmed that the selected loci had a significant effect on an animal’s weight in six days after birth.

In addition to the performed analysis, it was also estimated what body weight increase could be expected in the animals whose BV was included in the fourth (top) quartile (> 75 %). Student’s t-criterion was used to compare the mean values of the phenotype corrected for the abovementioned covariants for the whole cohort and the animals included in top BV quartile. It was found that the weight of the top-quartile animals was 1.8 % (around 55 grams, p-value = 0.67) higher than that in the averaged population. Thus, the selected markers can be potentially used for development of MAS test systems in sheep.

## Conclusion

The GWAS-MAP|ovis platform has been designed for aggregation, unification, storage and analysis of GWAS summary statistics in sheep. Today, it contains more than 34 million SNP associations for 80 different traits related to meat productivity in sheep. The platform enables one to search for information on associations for a particular SNP, plot Manhattan graphs and carry out colocalization analysis. The platform can be useful for scientists as an investigating tool to study sheep genetics and for breeders as a solution for searching for potential MAS loci.

## Conflict of interest

The authors declare no conflict of interest.
